# C‐reactive protein levels in patients with amyotrophic lateral sclerosis: A systematic review

**DOI:** 10.1002/brb3.2532

**Published:** 2022-02-24

**Authors:** Sanjeev Kharel, Rajeev Ojha, Veeramani Preethish‐Kumar, Riwaj Bhagat

**Affiliations:** ^1^ Department of Internal Medicine Maharajgunj Medical Campus Tribhuvan University Institute of Medicine, Maharajgunj Kathmandu Nepal; ^2^ Department of Neurology Tribhuvan University Institute of Medicine, Maharajgunj Kathmandu Nepal; ^3^ Department of Neurology Neurofoundation hospitals Salem Tamil Nadu India; ^4^ Department of Neurology Boston University Medical Center Boston Massachusetts USA

**Keywords:** amyotrophic lateral sclerosis, biomarker, C‐reactive protein

## Abstract

**Introduction:**

Amyotrophic lateral sclerosis (ALS) is a progressive neurodegenerative disease affecting cortical and spinal motor neurons. There is a lack of optimal biomarkers to diagnose and prognosticate the ALS patients. C‐reactive protein (CRP), an inflammatory marker, has shown promising results in ALS patients.

**Materials and methods:**

PubMed, Embase, and Google Scholar databases were searched from 2000 to June 1, 2021 for suitable studies showing the relationship between CRP and ALS. The concentration of CRP levels was assessed between ALS patients and controls. Further, end outcomes like ALS functional rating scale (ALSFRS‐R), survival status, and mortality risks were assessed in relation to CRP levels.

**Results:**

Eleven studies including five case–control, five cohorts, and one randomized control study were assessed. There were 2785 ALS patients and 3446 healthy controls. A significant increment in CRP levels among ALS patients in comparison with healthy controls were seen in most of the studies. ALSFRS‐R and disease progression were found to be significantly correlated with CRP levels. Overall accuracy of CRP in CSF was 62% described in a single study.

**Conclusion:**

Although CRP has shown promise as a prognostic biomarker, extensive cohort studies are required to assess its prognostic value and accuracy in diagnosing ALS taking into account the confounding factors.

## Graphical abstract

Amyotrophic lateral sclerosis (ALS) is a fatal neurodegenerative disease that affects cortical and spinal motor neurons. The underlying neuroinflammatory process in the pathogenesis of ALS has researchers interested in C‐reactive protein (CRP) as a possible disease biomarker. We used a comprehensive review to investigate the link between CRP levels and disease progression in ALS patients versus healthy controls. The concentration of CRP levels was assessed between ALS patients and controls. Further, end outcomes like ALS functional rating scale, survival status, and mortality risks were assessed in relation to CRP levels.

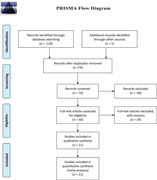

## 1 INTRODUCTION

Amyotrophic lateral sclerosis (ALS) is a fatal neurodegenerative disease that affects cortical and spinal motor neurons (Westeneng et al., 2018). Only 10% of ALS patients live 10 years or longer, with most dying within 3–5 years owing to respiratory arrest (Brown & Al‐Chalabi, 2017). The median incidence is 2.8 cases per 100,000 people per year, and the median prevalence is 5.4 cases per 100,000 people (Chiò et al., 2013). An ageing global population is predicted to increase ALS cases by roughly 70% from 2015 to 2040 (Arthur et al., 2016). Currently the clinical diagnosis of ALS is based on clinical and electrodiagnostic evidence as per the El Escorial criteria (Brooks et al., 2000).

The underlying neuroinflammatory process in the pathogenesis of ALS has researchers interested in C‐reactive protein (CRP) as a possible disease biomarker (Gordon, 2013). CRP is a sensitive and recognized systemic inflammatory marker generated by the liver in response to cytokines including interleukin‐6 (IL‐6), IL‐1, and tumor necrosis factor‐α (Mahmoud & Rivera, 2002). CRP is used as a chronic inflammatory measure in epidemiological investigations because it is readily available, reliable, and stable (Libby et al., 2009; Pearson et al., 2003). Regardless of its limitations, the ALS functional rating scale‐revised (ALSFRS‐R) score is used to track disease progression in ALS patients (Bacci et al., 2015). Precisely, some investigations indicated a strong association between CRP levels and ALFSRS‐R score, illness progression, and survival status in ALS patients, whereas others did not (Beers et al., 2020; De Schaepdryver et al., 2020; Huang et al., 2020; Keizman et al., 2009; Lunetta et al., 2017; Ryberg et al., 2010). We used a comprehensive review to investigate the link between CRP levels and disease progression in ALS patients versus healthy controls (HCs).

## 2 METHODS

### 2.1 Search methods and study selection

We identified studies from PubMed, Embase, and Google Scholar (from database search from 2000 to June 1, 2021). The search was limited to only English language. The following keywords or MeSH terms were used: *amyotrophic lateral sclerosis*, *motor neuron disease*, *Lou Gehrig's disease*, *ALS*, *MND*, *C‐reactive protein*, and *CRP*. In addition, references of selected studies were hand searched. The related abstracts and conference proceedings in journals and preprint servers were also searched where available. All the studies were selected and reviewed by two independent investigators (S. K. and R. O.). Any disagreements were resolved by the third author.

**FIGURE 1:**
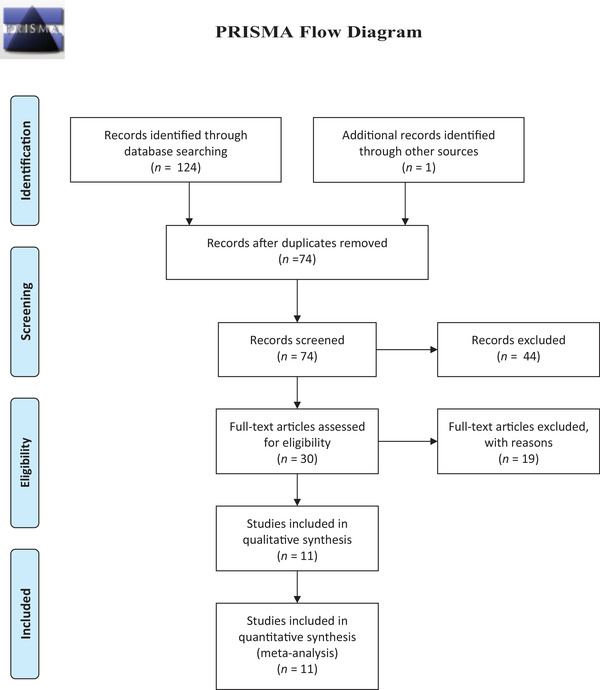
The diagram detailing our literature search and selection

The flowchart for the selection process is shown in Figure 1. This systematic review is reported according to the Preferred Reporting Items for Systematic Reviews and Meta‐Analyses (PRISMA) guidelines in conjugation with the PRISMA checklist and flow diagram, for manuscript format development (Liberati et al., 2009); see also Appendix S1.

### 2.2 Study inclusion and exclusion criteria

The inclusion criteria for selection of studies should have fulfilled the following criteria: (1) patients diagnosed with definite or probable ALS according to revised El Escorial criteria; (2) data showing relationship between serum/CSF CRP levels and disease status in ALS patients; (3) data with end point assessment like disease progression over time, ALSFRS‐R, survival outcome, or mortality risk; and (4) use of standardized method to quantify CRP levels.

The exclusion criteria were as follows: (1) animal or in vitro studies; (2) insufficient data; (3) duplicate article; (4) case reports, reviews, or meta‐analysis; (5) non‐English studies.

### 2.3 Data extraction

Data were extracted by two independent investigators (S. K. and R. O.) in an Excel spreadsheet (Microsoft Corp) using a standardized data extraction form, and results were compiled to complete the following fields: author, year of publication, study site, study design, number of patients (ALS and controls), age of patients, source of sample, CRP subtypes, CRP assays used, and end point assessment/main outcomes. A third reviewer (V. P. K.) was consulted to resolve inconsistencies when consensus could not be reached. In case of confusions and incomplete data, corresponding authors of the article were emailed.

### 2.4 Quality appraisal

Quality assessment for the included observational studies was performed using the Newcastle–Ottawa quality assessment scale, which consists of three domains: (a) selection of the participants; (b) comparability between the groups; and (c) ascertainment of the outcome in cohort studies. While for randomized controlled trial (RCTs) studies, the risk of bias was evaluated by the tool described in the Cochrane Handbook for Systematic Reviews of Interventions. (https://training.cochrane.org/handbook/current) Studies with scores greater than 5 (out of total 9) were included in our study. Two independent authors discussed and assessed the quality of studies.

**LIMITATION**

Although this review is first to describe the potential role of CRP levels in ALS patients for disease progression and survival status, several limitations should be noted. We could only include a single study assessing accuracy of CRP in ALS in CSF hindering the conclusion as diagnostic biomarker. The other limitation was inability to do meta‐analysis that could provide robust data. Another limitation was the exclusion of non‐English articles. Similarly, different samples, CRP subtypes, and cut‐off values were used in the study included in our review. Confounders like age, sex, and BMI were not addressed in many studies.

### 2.5 Outcome measures

The objective of review was to assess the CRP levels in ALS patients and compare it with the controls. In addition, the other objective was to establish CRP as a biomarker or prognostic marker through assessment of outcomes like ALSFRS‐R, Appel ALS (AALS) score, survival status, and mortality risks.

## 3 RESULTS

### 3.1 Literature search and data extraction

Initially, the search retrieved 124 articles, from which 74 articles remained after removal of duplicates. After screening the titles and abstract, we shortlisted 30 articles. Further, 19 articles were excluded according to predetermined inclusion and exclusion criteria. Therefore, 11 studies including 2785 ALS patients and 3446 HCs were included in our review. Figure 1 shows the results of our literature search and selection. The characteristics of each included study discussed below are summarized in Table 1.

**TABLE 1:** Detailed characteristics of studies included in our review

Authors	Year of publication	Study site	Study design	Number (patients/controls)	Age (patients/controls)	Sample source	CRP subtypes with cut‐off value	CRP assay type	Main outcomes/end point assessment
Keizman et al. (2009)	2009	Israel	Case–control	80/80	59 ± 19 years (range 25−88)	Serum	Wide‐range C‐reactive protein (0‐5 mg/L)	NA	ALS functional rating scale (ALSFRS‐R)
Ryberg et al. (2010)	2010	USA	Case–control	100/41	52.6/44.9 years	CSF	C‐reactive protein	ELISA	Mass spectral peaks, concentration of CRP levels
Miller et al. (2015)	2015	USA	RCT	94(NP001)/42(placebo)	54.4 (12.4)/53.7 (9.52) years	Plasma	Wide‐range C‐reactive protein (wr‐CRP)	NA	ALSFRS‐R, vital capacity
Nagel et al. (2017)	2017	Germany	Case–control	289/506	65.7 (10.5) years/66.3 (9.8) years	Serum	hs‐CRP (mg/L)	Latex‐enhanced high‐sensitivity immunonephelometry assay	Concentration of hs‐CRP, mortality/survival status
Lunetta et al. (2017)	2017	Italy	Cohort	394	60.18 (13.60) years	Serum	CRP (≤0.20 mg/dl)	NA	ALSFRS‐R, survival status,
Beers et al. (2020)	2020	USA	Cohort (first group)	68/55	58.8 (1.57)/57.6 (2.15) years	Serum	C‐reactive protein	ELISA	Appel ALS (AALS) score
			Cohort (second group)	100/60	62.6 (1.47)/63.5 (1.15)	Serum	C‐reactive protein	ELISA	Appel ALS (AALS) score
Chełstowska & Kuźma‐Kozakiewicz (2020)	2020	Poland	Cohort	203	mean: 56 years; median:57 years	Serum	C‐reactive protein	NA	Concentration of CRP levels, ALSFRS‐R
Cui et al. (2020)	2020	Sweden	Case–control	525/2625	65.90 ± 13.10/65.87 ± 13.09 years	Serum	High‐sensitivity C‐reactive protein	Behring nephelometer and reagent	Change in CRP levels
De Schaepdryver et al. (2020)	2020	Belgium, Italy	Cohort	383	NA	Serum	C‐reactive protein		ALSFRS‐R and survival status
Huang et al. (2020)	2020	USA	Case–control	108/79		Plasma, CSF	C‐reactive protein	MSD (Meso Scale Discovery) V‐Plex	Disease progression ALSFRS‐R, slow vital capacity
Sun et al. (2020)	2020	Sweden	Cohort	399	66.25 years	Serum	hs‐CRP (mg/L)	NA	Mortality risk

Among the selected literature, five were cohort studies (Beers et al., 2020; Chełstowska & Kuźma‐Kozakiewicz, 2020; De Schaepdryver et al., 2020; Lunetta et al., 2017; Sun et al., 2020), five were case–control studies (Cui et al., 2020; Huang et al., 2020; Keizman et al., 2009; Nagel et al., 2017; Ryberg et al., 2010), and only one was RCT (Miller et al., 2015). The various dates of publication ranged between 2009 and 2020. The mean age of ALS patients was in the range of 52.6 to 66.25 years, whereas the age of controls spanned from 44.9 to 66.3 years. The different “CRP assay” type and end point assessment outcomes used in the study are described in Table 1. The study by Beers et al. (2020) had data on two cohort (nested case control in each cohort) groups.

### 3.2 Quality assessments

The New‐Castle Ottawa Scale ranges from 6 to 8. All the studies were included in our analysis (Beers et al., 2020; Chełstowska & Kuźma‐Kozakiewicz, 2020; Cui et al., 2020; De Schaepdryver et al., 2020; Huang et al., 2020; Keizman et al., 2009; Lunetta et al., 2017; Nagel et al., 2017; Ryberg et al., 2010; Sun et al., 2020). The RCT included in our study (Miller et al., 2015) showed low risk of biases in the random sequence generation, allocation concealment, selective reporting, blinding of participants and personnel, and blinding of outcome assessment (see also Appendix S2).

### 3.3 Use of CRP

Different types of CRP markers with their own cut‐off value investigated by different assays from different sample sources were used in the studies. Three studies used high sensitivity CRP (Cui et al., 2020; Nagel et al., 2017; Sun et al., 2020), two studies used wide‐range CRP (Keizman et al., 2009; Miller et al., 2015), while remaining utilized standard CRP markers (Beers et al., 2020; Chełstowska & Kuźma‐Kozakiewicz, 2020; De Schaepdryver et al., 2020; Huang et al., 2020; Lunetta et al., 2017; Ryberg et al., 2010). Samples from eight studies were from serum (Beers et al., 2020; Chełstowska & Kuźma‐Kozakiewicz, 2020; Cui et al., 2020; De Schaepdryver et al., 2020; Keizman et al., 2009; Lunetta et al., 2017; Nagel et al., 2017; Sun et al., 2020), one from plasma (Miller et al., 2015), one from CSF (Ryberg et al., 2010), and the one from both plasma and CSF (Huang et al., 2020). ELISA, nephelometer, and MSD (Meso Scale Delivery) V‐plex were the CRP assays used (Table 1).

### 3.4 Main outcomes/end point assessment

All the included studies had at least one of the following outcomes/end points: concentration of CRP levels, changes in CRP levels, disease progression over time measured as ALSFRS‐R or AALS score, survival status, and mortality risk.

### 3.5 CRP levels among ALS patients and controls

There were five case–control studies that compared the CRP levels among ALS patients and HCs. Keizman et al. (2009) showed significant increase in wide‐range CRP levels in repeated blood tests (three examinations in the interval of 3 months) among ALS patients when compared with controls. Similar study by Ryberg et al. (2010) showed statistically significant increase of CRP levels in CSF of ALS patients (11.24 ± 1.52 ng/ml) than controls (5.84 ± 1.01 ng/ml). Population‐based ALS registry of Germany showed no difference in high‐sensitivity CRP concentrations between ALS cases [median:1.29 (0.64, 3.22)] and controls [median: 1.14 (0.65, 2.80)] (Miller et al., 2015). Beers et al. (2020) described results from two cohorts from subpopulation. In first cohort, CRP was elevated in the sera of fast progressing patients compared with HCs (*p* = .006); however, no difference was seen among slow progressing patients and HCs (*p* = .075). Overall patients (fast and slow progressing) had elevated CRP compared with HCs (*p* < .008). In second cohort, all ALS patients (fast and slow progressing) CRP was elevated compared with HCs (*p* < .001). Huang et al. (2020) also showed no significant increment of CRP in ALS patients when compared with controls (*p* = .76); however, increasing trend was noted among the C9orf72 positive ALS patients. The pooled frequency analysis showed 53% of ALS patients had higher level of CRP when compared with the HCs.

A study by Cui et al. (2020) showed the temporal distribution of CRP levels among ALS patients and HCs. There was slightly lower levels and higher levels of CRP, 2 years before and after the diagnosis of ALS, respectively. No change in CRP level was noted among HCs when followed through 4 years.

### 3.6 CRP levels as diagnostic biomarker in ALS patients

Ryberg et al. (2010) was the only study that assessed the accuracy of CRP levels as a diagnostic biomarker. They showed CRP ELISA overall accuracy of 62% (sensitivity of 51% and specificity of 85%) to discriminate ALS from HCs using a cut‐off value of 9 ng/ml. The CRP mass peak showed an overall accuracy of 62% (sensitivity of 65% and specificity of 60%) to differentiate ALS from all non‐ALS cases.

### 3.7 CRP levels as prognostic biomarker in ALS patients

There were seven studies that assessed CRP as a prognostic biomarker as compared with the disease progression (ALSFRS‐R and AALS score) and survival/mortality rate. Only five studies showed positive association between CRP levels and disease progression/survival rate.

Chełstowska & Kuźma‐Kozakiewicz (2020) studied biochemical parameters of 203 ALS patients to assess their nutritional status. Among 20% of patients who had biochemical features of inflammation, CRP was elevated in only 5.9% patients. Higher number of cases (9.8%) with inflammation progressed to severe dysphasia as per ALSFRS subscore requiring enteral feeding.

Keizman et al. (2009) showed a significant correlation between the ALSFRS‐R and the wide‐range CRP (*p* < .001). They also stated that high levels of wide‐range CRP were observed as disease progressed. Similarly, logistic regression analysis showed OR (3.25, *p* < .001) for each unit increase in wide‐range CRP. The CRP level was also found to be a valuable preclinical predictor for the subsequent development of an overt respiratory tract infection in this study.

Lunetta et al. (2017) conducted a large multicentric cohort study from Italy. The study found serum CRP levels in Neuromuscular Omnicentre (NEMO) cohort to be inversely correlated with severity of functional impairment as measured by ALSFRS‐R (*r* = −0.14818; *p* = .004) at initial evaluation and after 1 year follow‐up. The study included 50 patients of NEMO cohort and they also proposed that the correlation was significant when age, sex, body mass index (BMI), and smoking status were adjusted. Similarly, serum CRP levels were correlated with patient survival in NEMO group (hazard ratio, 1.129; 95%CI, 1.033–1.234; *p* = .007) and independent cohort groups (hazard ratio, 1.044; 95%CI, 1.016–1.056; *p* = .001).

Beers et al. (2020) studied two nonoverlapping cohorts of patients and controls; first cohort (ALS patients = 68, controls = 55) and second cohort (ALS patients = 100, controls = 60). They assessed the disease burden by AALS scoring system and defined fast and slow progressors as progression rate of >= and <= 1.5 AALS points/month, respectively. They showed serum CRP positively correlated with the patient's burden of disease (*p* < .001, r = 0.420) and patient's disease progression rate (*p* < .001, *r* = 0.817) in the second cohort of patients.

In a study by Sun et al. (2020), they found patients with a higher than median level of log CRP (HR 1.33; 95% CI 1.04−1.71) had high risk of mortality. Elevated levels of CRP were found in very fast (death with 1 year of diagnosis) and medium progression (death within 1–3 years of diagnosis) groups compared with the slow progression (death after 3 years of diagnosis) group.

There were two negative studies that showed no association between CRP levels and survival rate.

A study by De Schaepdryver et al. (2020) conducted in two cities, Belgium and Italy, showed no association of serum CRP with survival rate (HR 0.86, 95% CI 0.61 to 1.23, *p* = .4196). A population‐based registry study in Germany by Nagel et al. (2017) showed contrasting results. There was no significant association between hs‐CRP levels and longer survival. Furthermore, when analyzed by age and sex adjusted, no association of hs‐CRP levels and mortality was observed. Huang et al. (2020) studied the disease progression rate using inflammatory markers other than CRP levels.

## 4 DISCUSSION

To our knowledge this is the first systematic review including 11 studies; done to clarify concentration of CRP levels among ALS patient's versus HCs and CRP as a diagnostic and prognostic biomarker. Our study suggested 53% of ALS patients when compared with HCs had statistically significant elevated CRP levels. One study, Ryberg et al. (2010) assessed the accuracy of CRP level as a diagnostic marker in CSF. Finally, majority of the studies (five out of seven) showed elevated CRP levels as a prognostic biomarker.

Familial ALS is linked with the common genetic variants including Cu/Zn superoxide dismutase‐led oxidative stress, and transactive response DNA‐binding protein of 43 kDa (TARDBP), fused in sarcoma and C9orf72 induced RNA processing defect, whereas both genetic and environmental factors have been linked to sporadic ALS. (Gordon, 2013) Protein misfolding, free radical production, excitotoxicity, axonal transport disruption, mitochondrial malfunction, and inflammation are thought to contribute to cell death (Gordon, 2013). Inflammatory process is thought to play two roles in ALS where adaptive and innate immune responses promote neuroprotection or neurotoxicity depending on the illness stage through uncontrolled recruitment of the microglial and other immune cells (Philips & Robberecht, 2011; Zhao et al., 2013).

Several inflammatory biomarkers apart from CRP, like serum Cystatin C and transtyrectin (Keizman et al., 2009), serum‐soluble CD14 (Beers et al., 2020), and serum apokines (Nagel et al., 2017), were found to be raised in ALS patients compared with the HCs. (Beers et al., 2020; Nagel et al., 2017; Ryberg et al., 2010). Likewise, other inflammatory markers mainly cytokins and glial surface products were seen to be raised in the ALS patients (Cui et al., 2020; De Schaepdryver et al., 2020; Huang et al., 2020; Sun et al., 2020). Cui et al. (2020) eloquently showed the temporal distribution of CRP levels among ALS patients and HCs, where lower‐level CRP raised after the diagnosis of ALS, whereas it remained at lower level in the HCs. This strongly suggests the underlying neuroinflammatory process in the pathogenesis of ALS. It is postulated that ALS‐associated immune responses present first as an activation of glial cells of CNS before presenting in the peripheral nervous system (McCauley & Baloh, 2019). CRP, a non‐specific systemic inflammatory marker, might be elevated in response to the elevated cytokines in ALS patients (McCauley & Baloh, 2019). High levels of CRP enhance the permeability of the blood–brain barrier, which in turn activates brain microglia, resulting in a vicious cycle (Closhen et al., 2010; Hsuchou et al., 2012). This might explain the higher CRP levels with the disease progression. Nevertheless, it is also thought that respiratory infections may be the underlying cause for rapid rise in CRP during months before death (Cui et al., 2020). Beers et al. (2020) found in their first cohort of ALS patients, the fast progressors have statistically significant elevated CRP levels compared with HCs, whereas slow progressors did not. This suggests robust inflammatory process might occur in the fast progressors compared with the slow progressors.

Yet, search for the ideal biomarker for the ALS is a quest. An ideal biomarker for ALS should be sensitive and specific with easy accessibility and applicability for all ALS patients regardless of their physical status. The biomarker should be able to diagnose ALS before onset of symptom, predict disease progression, and differentiate ALS from other clinically similar neurodegenerative disease (Turner et al., 2009). Majority of the studies that studied CRP levels in ALS patients were from blood, suggesting its easy accessibility (Beers et al., 2020; Chełstowska & Kuźma‐Kozakiewicz, 2020; Cui et al., 2020; De Schaepdryver et al., 2020; Huang et al., 2020; Keizman et al., 2009; Nagel et al., 2017; Sun et al., 2020). Ryberg et al. (2010) determined overall accuracy of CRP by ELISA technique to be 62% (sensitivity of 51% and specificity of 85%); however, CRP was drawn from the CSF. The utility of CRP as a biomarker could not be made based on a single study. We are still uncertain about its accuracy from the blood sample. There are several confounding factors while interpreting the CRP level, which might be affected by chronic diseases, cardiovascular risk factors, BMI, recent infection, surgery, fracture, stroke, and inflammation sensitive drugs (Turner, Kiernan, Leigh & Talbot, 2009). Other sensitive and specific inflammatory molecules, metabolic markers, and neurofilaments are the best candidates for biomarker for ALS patients (Costa & de Carvalho, 2016). Further, ALS is a neurodegenerative disease commonly in middle age population and patients in those age group often suffer from other comorbid condition, can influence CRP level.

A RCT by Miller et al. (2015) assessing the safety, tolerability, and preliminary efficacy of NP001, a novel immune regulator, showed slowing of progression of disease in high‐dose group patients treated with NP001 with higher wide‐range CRP levels compared with patients with normal baseline CRP. In another study, CRP ultrasensitive was significantly elevated (*p* = .0036) in ALS patients requiring noninvasive ventilation and tracheostomy‐invasive ventilation compared with intake clinic patients while was also correlated with ALSFRS‐Rt (*p* = .0018). As CRP levels were significantly (*p* = .0313) reduced after riluzole therapy, it may be considered as a potential biomarker for treatment responsiveness. (Sanjak, 2018) Currently, our review suggests, CRP can be used as an easily accessible prognostic biomarker for the ALS diagnosis, especially among the fast progressor but lacks behind in assessing the accuracy of serum CRP level in the diagnosis of the ALS especially in serum samples.

## 5 CONCLUSION

Our review suggested that CRP is a reliable prognostic biomarker of ALS that correlated with the disease progression and therapeutic response. CRP was elevated in majority of the ALS cases compared with the HCs. Further studies are needed for assessment of the accuracy of serum CRP levels in the diagnosis of ALS taking in account of the confounding factors.

## CONFLICT OF INTEREST

The authors report no conflicts of interest. The authors alone are responsible for the content and writing of the paper.

## FUNDING

None.

### PEER REVIEW

The peer review history for this article is available at https://publons.com/publon/10.1002/brb3.2532

## Supporting information




**Appendix 1**: Search strategy used in the current systematic review.
**Appendix 2**: Quality assessment of the included observational articles.

## Data Availability

Data will be available on request from the authors.
