# Supplementary material for: C‐reactive protein levels in patients with amyotrophic lateral sclerosis: A systematic review
Source: Brain Behav. 2022 Feb 24;12(3):e2532. doi: 10.1002/brb3.2532 (PMC8933772; doi:10.1002/brb3.2532)
Supplement: Supplementary file 1 — Appendix 1: Search strategy used in the current systematic review. Appendix 2: Quality assessment of the included observational articles. [file BRB3-12-e2532-s001.docx]

Supplementary Content

**C reactive protein levels in patients with amyotrophic lateral sclerosis: A systematic review**

**Appendix 1:** Search strategy used in the current systematic review.

**Appendix 2:** Quality assessment of the included observational articles.

This supplementary material has been provided by the authors to give readers additional information.

**Appendix 1:** Search strategy used in the current systematic review and meta- analysis.

For PubMed

#1: "Amyotrophic Lateral Sclerosis"[Mesh] OR "ALS" OR "Motor Neuron Disease" OR "Lou Gehrig Disease"

#2: "C-Reactive Protein"[Mesh] OR "CRP"

#3: #1 AND #2

Filters: human subjects, English, Time frame from 2000 to 2021/06/01

# Final search strategy after filters:

# (("Amyotrophic Lateral Sclerosis"[MeSH Terms] OR "ALS"[All Fields] OR "Motor Neuron Disease"[All Fields] OR "Lou Gehrig Disease"[All Fields]) AND ("C-Reactive Protein"[MeSH Terms] OR "CRP"[All Fields])) AND (2000:2021[pdat])

# Total=44

**Appendix 2:** Quality assessment of the included article

| Study Name | Selection | Comparability | Outcome | Total Score | Risk of Bias | Included/ Excluded |
| --- | --- | --- | --- | --- | --- | --- |
| **Case-Control Studies** |  | | | | | |
| Keizman et al. | 3 | 2 | 2 | 7 | Low | Included |
| Ryberg et al. | 3 | 2 | 2 | 7 | Low | Included |
| Nagel et al. | 4 | 2 | 2 | 8 | Low | Included |
| Huang et al. | 3 | 2 | 2 | 7 | Low | Included |
| Cui et al. | 3 | 2 | 2 | 7 | Low | Included |
| **Cohort Studies:** |  |  |  |  |  |  |
| Lunetta et al. | 3 | 2 | 3 | 8 | Low | Included |
| Beers et al. | 4 | 2 | 2 | 8 | Low | Included |
| Chelstowska et al. | 3 | 2 | 1 | 6 | Low | Included |
| Schaepdryver et al. | 3 | 2 | 2 | 7 | Low | Included |
| Sun et al. | 4 | 2 | 2 | 8 | Low | Included |

# Note: Mean scores greater or equal to 5 are included in analysis.
